# Operation for Procidentia Uteri

**Published:** 1871-08

**Authors:** C. C. F. Gay

**Affiliations:** Surgeon to the Buffalo General Hospital


ART. III.—*Operation for Procidentia Uteri.* By C. C. F. GAY, M. D., Surgeon to the Buffalo General Hospital.

———

Mrs. Rosanna G., aged 35 years, has had procidentia complete for 13 years. Has borne five children, three of whom were born since the occurrence of procidentia. The os protrudes two or two and a half inches beyond the vulva. Treatment, preliminary to operation, consisted of application of weak solution of nitrate of silver to the prolapsed surface of the vaginal walls, and the administration of a dose of castor oil the evening previous to the operation.

On June 6th, 1871, I operated in the following manner, there being present Drs. Gould, Boardman, Barnes, Chace, Willoughby and McNeal, and Mr. Bartow, medical student. Chloroform was administered, and the woman placed in the semi-prone position upon a table, with the limbs flexed. Sims' speculum was used.

I ante-verted the uterus as recommended by Dr. Emmett, in order to keep the os well up out of the way, but notwithstanding, found the uterine holder in the hands of an assistant of service.

The mucous membrane in front of the os was now seized, and caught up by the tenacula, one in each hand, at two points, and brought together at the centre, when it was ascertained that a strip four inches in length could be denuded, and brought in apposition without causing much tension.

One of the tenacula was dropped, and the other, held by the hand, caught in the mucous membrane, and a strip denuded three-quarters of an inch in length, in line with the axis of the vagina, by one half inch in width. This completed, the other tenaculum was taken up and the same amount of surface denuded. These two raw surfaces were now connected by denuding a strip of mucous membrane one half inch wide across and in front of the os, making a denuded surface upon the anterior wall of the vagina, and in front of the os, from four inches to four inches and a half in length.

This raw surface was now brought together by introducing two silver sutures with a running stitch, one suture passing along the upper margin and the other the lower margin. The wire was drawn through with the silk loop, the parts brought together and the wire twisted. In this respect I did not follow Emmett, as it will be recollected that he uses but a single suture in this part of the operation. I am not able to say that there is any advantage in using the two instead of one suture, but it would appear that when two are used, the parts ought to be doubly secure. In finishing the operation I again departed from the plan recommended, not indeed, so much from choice, as from compulsion, in consequence of the peculiar conformation of the vaginal walls, and on account also of the position of the redundant mucous membrane, which prolapsed. My incisions did not diverge from a point just above the meatus and run up so as to connect with the horizontal strip, but when completed the surface denuded was shaped like the letter **U**, the convexity looking toward the vaginal outlet, and did not reach down so nearly to the meatus by three-quarters of an inch as in Emmett's operation, and the two lines of denuded surface were nearly parallel, thus it will be seen that much more tissue would be taken up and enclosed within this space when the raw surfaces were brought together. I found one advantage in this, not so many sutures were required as would have been necessary had the incisions ran down to the meatus. Seven or eight sutures were used, whereas, had the denudation extended down to a point, at least three more sutures would have been required.

The parts were approximated, the sutures twisted, cut off one inch in length, and turned upward. On the eleventh day the sutures were removed and union found to have taken place throughout the entire extent of my scarifications. On the 23d of June the patient was setting up in bed. I made digital examination, and cannot hesitate to say that the success of the operation is complete.

Just sufficient opium was given to constipate the bowels, and the bladder was twice or thrice daily evacuated by the catheter. Made examination July 5th, there is no further prolapse, and the woman is walking about her room, and up and down stairs.

Case II.—Mrs. Joice, aged 42 years, has borne six children. The procidentia is complete, having existed for one year, and was caused, she says, by coughing, Preliminary treatment, which consisted of local application of nitrate silver grs. ij. to aqua ℥i. daily, was continued for ten days.

On August 5th, 1871, under the influence of chloroform the operation was performed for radical cure. The woman was placed upon a small table in the semi-prone position, and the speculum of Sims used. The operation was done in all essential respects like the former, only the first suture was doubled, as used by Emmett, instead of using two single sutures as in the former case reported. It being so very tedious to introduce the first suture in a running stitch through so long a surface, it, of course, is desirable to dispense with one of them, provided the double suture will answer as well. The portion of mucus membrane denuded near the meatus was circular and not pointed. The mucous membrane was very tough, requiring a sharp pointed needle to penetrate easily. There was not much hemorrhage, and all the cutting was done with the double curved scissors.

Nine sutures in all were used. On August 15th, the eleventh day after the operation, the sutures were removed, and firm union obtained. The floor upon which the os uteri may rest feels firm and strong as a good sized rope, and it will be impossible, without very considerable vaginal dilation, for the uterus or vaginal walls again to prolapse. ***

---

***Note.—A report of these and similar cases, made a year or two later would be of value; time is required before results in such cases can be properly estimated. Dr. West, in his work on diseases of women, speaking on this subject, says: "A verdict not more favorable must be pronounced on an operation, which has sometimes been practiced, which consists in the endeavor to contract the vaginal canal, either by the removal of strips of the mucous membrane, or by the use of actual cautery, or of strong caustics, so as to produce cicatrices on its walls, and consequent shrinking of its calibre, or by the insertion of sutures in a peculiar manner, with the view of obtaining a similar result. The objection, and to my mind, the fatal objection, to these as to the other surgical proceedings for the cure of prolapsus uteri, is furnished not merely by the imperfect nature of the cure which they accomplish, and the new discomforts and inconveniences which they substitute for those before experienced, but still more by the want of permanence in their result, even when their issue is most fortunate, and this objection seems to me all the more serious, since failure in this respect appears to be the rule, success the rare exception. I think, too, that if we consider the circumstance in which prolapsus, either of the uterus, rectum or bladder takes place, we can scarcely expect that the result of the operation should be other than temporary; that the cicatrix tissues should yield to the pressure from above, and all their other causes remaining unremoved, misplacement of the organs should in most instances recur. The operations already referred to, seemed to deserve rejection rather on account of their inadequacy to effect a permanent cure of the evils for the removal of which they have been suggested, than on account of the great difficulties or great danger of their performance."—Ed.

